# Supplementary material for: Deletion of wfs1 Impairs Oligodendrocyte Precursor Cells Dorsal Distribution and Myelination Through the wfs1-hmgcs1 Axis in Zebrafish
Source: Biology (Basel). 2026 Aug 16;15(16):1402. doi: 10.3390/biology15161402 (PMC13510151; doi:10.3390/biology15161402)
Supplement: Supplementary file 1 [file biology-15-01402-s001.zip › File S1-Transcriptome sequencing data and differentially expressed genes (DEGs) in wfs1-- zebrafish at 5 dpf/1.GO/wfs1KOvsWT/GSEA/wfs1KO/NUCLEAR_TRANSPORT/NUCLEAR_TRANSPORT(GO_0051169).html]

Details for gene set NUCLEAR\_TRANSPORT(GO:0051169)[GSEA]


|  || Dataset | deg.sample |
| Phenotype | sample.cls |
| Upregulated in class | wfs1KO |
| GeneSet | NUCLEAR\_TRANSPORT(GO:0051169) |
| Enrichment Score (ES) | 0.3059878 |
| Normalized Enrichment Score (NES) | 1.8243945 |
| Nominal p-value | 0.0 |
| FDR q-value | 0.11496642 |
| FWER p-Value | 0.604 |
Table: GSEA Results Summary

  

Fig 1: Enrichment plot: NUCLEAR\_TRANSPORT(GO:0051169)
  
Profile of the Running ES Score & Positions of GeneSet Members on the Rank Ordered List

  

| PROBE | DESCRIPTION   (from dataset) | GENE SYMBOL | GENE\_TITLE | RANK IN GENE LIST | RANK METRIC SCORE | RUNNING ES | CORE ENRICHMENT || 1 | 266985 | npm1a |  |  | 83 | 4.783 | 0.1047 | Yes |
| 2 | 393973 | rae1 |  |  | 554 | 2.834 | 0.1535 | Yes |
| 3 | 406860 | ipo9 |  |  | 795 | 2.389 | 0.1995 | Yes |
| 4 | 407710 | xpo7 |  |  | 1448 | 1.693 | 0.2170 | Yes |
| 5 | 563448 | zgc:193690 |  |  | 1628 | 1.584 | 0.2469 | Yes |
| 6 | 566321 | si:ch73-182a11.2 |  |  | 1882 | 1.422 | 0.2708 | Yes |
| 7 | 333980 | xpo6 |  |  | 2292 | 1.251 | 0.2860 | Yes |
| 8 | 406343 | kpna7 |  |  | 2896 | 1.059 | 0.2908 | Yes |
| 9 | 492509 | pcid2 |  |  | 3131 | 1.006 | 0.3060 | Yes |
| 10 | 768163 | chtopb |  |  | 6462 | 0.540 | 0.2133 | No |
| 11 | 324743 | snupn |  |  | 6714 | 0.518 | 0.2171 | No |
| 12 | 337461 | nup35 |  |  | 6734 | 0.516 | 0.2280 | No |
| 13 | 436607 | kpna2 |  |  | 7746 | 0.439 | 0.2061 | No |
| 14 | 321786 | pttg1ipa |  |  | 7785 | 0.438 | 0.2147 | No |
| 15 | 767721 | uhmk1 |  |  | 7801 | 0.437 | 0.2240 | No |
| 16 | 559102 | rangrf |  |  | 8140 | 0.416 | 0.2227 | No |
| 17 | 562607 | tnpo1 |  |  | 9354 | 0.344 | 0.1923 | No |
| 18 | 100319138 | ipo13 |  |  | 9639 | 0.324 | 0.1906 | No |
| 19 | 394115 | ranbp3a |  |  | 9851 | 0.313 | 0.1910 | No |
| 20 | 100002393 | zgc:175066 |  |  | 10021 | 0.302 | 0.1925 | No |
| 21 | 445231 | nup85 |  |  | 10935 | 0.248 | 0.1693 | No |
| 22 | 406510 | nup58 |  |  | 11164 | 0.235 | 0.1674 | No |
| 23 | 568298 | pom121 |  |  | 11330 | 0.226 | 0.1673 | No |
| 24 | 570158 | alkbh5 |  |  | 11439 | 0.220 | 0.1688 | No |
| 25 | 192325 | ncbp2 |  |  | 11875 | 0.197 | 0.1595 | No |
| 26 | 393917 | ddx39b |  |  | 12078 | 0.186 | 0.1573 | No |
| 27 | 100007012 | si:dkeyp-97b10.3 |  |  | 12184 | 0.180 | 0.1581 | No |
| 28 | 553305 | ipo4 |  |  | 12217 | 0.178 | 0.1611 | No |
| 29 | 30707 | cse1l |  |  | 12382 | 0.170 | 0.1597 | No |
| 30 | 368640 | kpna3 |  |  | 12628 | 0.158 | 0.1556 | No |
| 31 | 100529857 | nup214 |  |  | 13495 | 0.115 | 0.1309 | No |
| 32 | 571089 | tpra |  |  | 14107 | 0.087 | 0.1136 | No |
| 33 | 393663 | ranbp3b |  |  | 14333 | 0.077 | 0.1083 | No |
| 34 | 445035 | thoc7 |  |  | 14346 | 0.076 | 0.1096 | No |
| 35 | 321491 | chtopa |  |  | 14836 | 0.054 | 0.0954 | No |
| 36 | 323865 | nxf1 |  |  | 14875 | 0.052 | 0.0954 | No |
| 37 | 322969 | nup50 |  |  | 14954 | 0.049 | 0.0940 | No |
| 38 | 777756 | nup98 |  |  | 15327 | 0.031 | 0.0830 | No |
| 39 | 386850 | eif6 |  |  | 15361 | 0.030 | 0.0827 | No |
| 40 | 445204 | nutf2l |  |  | 15430 | 0.026 | 0.0811 | No |
| 41 | 558395 | xpo1a |  |  | 15460 | 0.024 | 0.0807 | No |
| 42 | 321739 | ipo7 |  |  | 15752 | 0.009 | 0.0718 | No |
| 43 | 558883 | tprb |  |  | 16254 | -0.009 | 0.0562 | No |
| 44 | 324551 | zc3h11a |  |  | 17190 | -0.040 | 0.0277 | No |
| 45 | 563392 | nup62l |  |  | 17218 | -0.042 | 0.0278 | No |
| 46 | 286746 | sdad1 |  |  | 17276 | -0.044 | 0.0270 | No |
| 47 | 562350 | nup107 |  |  | 17374 | -0.049 | 0.0250 | No |
| 48 | 100149852 | kpna6 |  |  | 17422 | -0.051 | 0.0247 | No |
| 49 | 432373 | ipo11 |  |  | 17779 | -0.066 | 0.0150 | No |
| 50 | 571621 | nup153 |  |  | 17848 | -0.069 | 0.0144 | No |
| 51 | 100317326 | si:ch1073-55a19.2 |  |  | 17968 | -0.074 | 0.0123 | No |
| 52 | 445302 | thoc3 |  |  | 18025 | -0.077 | 0.0122 | No |
| 53 | 335907 | anp32a |  |  | 18033 | -0.078 | 0.0138 | No |
| 54 | 445499 | tnpo2 |  |  | 18041 | -0.078 | 0.0153 | No |
| 55 | 327069 | cpsf6 |  |  | 18096 | -0.081 | 0.0154 | No |
| 56 | 393125 | nup155 |  |  | 18319 | -0.090 | 0.0104 | No |
| 57 | 561138 | xpo1b |  |  | 18716 | -0.109 | 0.0004 | No |
| 58 | 569455 | kpnb3 |  |  | 18740 | -0.110 | 0.0022 | No |
| 59 | 541444 | nmd3 |  |  | 18754 | -0.111 | 0.0043 | No |
| 60 | 751108 | nkd1 |  |  | 18935 | -0.119 | 0.0013 | No |
| 61 | 64605 | htatip2 |  |  | 19022 | -0.122 | 0.0013 | No |
| 62 | 445408 | gle1 |  |  | 19522 | -0.144 | -0.0111 | No |
| 63 | 436700 | eny2 |  |  | 19586 | -0.147 | -0.0098 | No |
| 64 | 436587 | ltv1 |  |  | 19725 | -0.154 | -0.0107 | No |
| 65 | 327373 | kpna4 |  |  | 20389 | -0.182 | -0.0275 | No |
| 66 | 572542 | kpna1 |  |  | 20404 | -0.183 | -0.0238 | No |
| 67 | 30572 | ran |  |  | 20453 | -0.185 | -0.0212 | No |
| 68 | 555520 | ipo8 |  |  | 20608 | -0.194 | -0.0217 | No |
| 69 | 394062 | tnpo3 |  |  | 20656 | -0.196 | -0.0187 | No |
| 70 | 553507 | npm1b |  |  | 20722 | -0.200 | -0.0163 | No |
| 71 | 436588 | polr2d |  |  | 21291 | -0.230 | -0.0290 | No |
| 72 | 325064 | thoc5 |  |  | 21379 | -0.235 | -0.0265 | No |
| 73 | 100536140 | poldip3 |  |  | 21465 | -0.239 | -0.0238 | No |
| 74 | 436589 | mcm3ap |  |  | 21641 | -0.248 | -0.0237 | No |
| 75 | 559225 | nup188 |  |  | 21691 | -0.251 | -0.0196 | No |
| 76 | 570700 | kpnb1 |  |  | 21734 | -0.253 | -0.0153 | No |
| 77 | 337399 | rrs1 |  |  | 21833 | -0.258 | -0.0126 | No |
| 78 | 560127 | alyref |  |  | 22014 | -0.268 | -0.0122 | No |
| 79 | 570926 | lzts2a |  |  | 22187 | -0.278 | -0.0114 | No |
| 80 | 402875 | dzip1 |  |  | 22233 | -0.280 | -0.0066 | No |
| 81 | 567236 | si:dkeyp-20g2.3 |  |  | 22976 | -0.326 | -0.0226 | No |
| 82 | 373077 | thoc1 |  |  | 23005 | -0.328 | -0.0161 | No |
| 83 | 100005471 | lzts2b |  |  | 23135 | -0.336 | -0.0126 | No |
| 84 | 406324 | abce1 |  |  | 23174 | -0.339 | -0.0062 | No |
| 85 | 368417 | thoc2 |  |  | 23234 | -0.343 | -0.0004 | No |
| 86 | 393863 | heatr3 |  |  | 23272 | -0.346 | 0.0062 | No |
| 87 | 406852 | nup133 |  |  | 23405 | -0.355 | 0.0100 | No |
| 88 | 558020 | xpot |  |  | 23442 | -0.357 | 0.0169 | No |
| 89 | 445490 | phax |  |  | 23901 | -0.386 | 0.0111 | No |
| 90 | 406586 | fam53b |  |  | 23904 | -0.386 | 0.0197 | No |
| 91 | 192339 | ddx19 |  |  | 24093 | -0.399 | 0.0228 | No |
| 92 | 751738 | nxt2 |  |  | 24134 | -0.402 | 0.0305 | No |
| 93 | 449768 | pttg1ipb |  |  | 24158 | -0.404 | 0.0388 | No |
| 94 | 751719 | zgc:153681 |  |  | 24257 | -0.411 | 0.0450 | No |
| 95 | 322269 | shfm1 |  |  | 24451 | -0.417 | 0.0483 | No |
| 96 | 378454 | aaas |  |  | 24595 | -0.421 | 0.0532 | No |
| 97 | 554106 | sarnp |  |  | 24867 | -0.441 | 0.0546 | No |
| 98 | 553205 | kpna5 |  |  | 25088 | -0.453 | 0.0578 | No |
| 99 | 751671 | rpain |  |  | 25348 | -0.471 | 0.0602 | No |
| 100 | 325550 | ddx39aa |  |  | 25415 | -0.476 | 0.0688 | No |
| 101 | 321744 | nup88 |  |  | 26614 | -0.589 | 0.0443 | No |
| 102 | 323464 | lsg1 |  |  | 27726 | -0.705 | 0.0252 | No |
| 103 | 30172 | nup93 |  |  | 28090 | -0.755 | 0.0307 | No |
| 104 | 550239 | hikeshi |  |  | 28529 | -0.827 | 0.0355 | No |
| 105 | 449827 | nutf2 |  |  | 30331 | -1.405 | 0.0103 | No |
| 106 | 324342 | xpo4 |  |  | 30757 | -1.724 | 0.0356 | No |
Table: GSEA details
[plain text format]

  

Fig 2: NUCLEAR\_TRANSPORT(GO:0051169)
  
Blue-Pink O' Gram in the Space of the Analyzed GeneSet

  

Fig 3: NUCLEAR\_TRANSPORT(GO:0051169): Random ES distribution
  
Gene set null distribution of ES for
 **NUCLEAR\_TRANSPORT(GO:0051169)**
